# Normal ranges for fetal electrocardiogram values for the healthy fetus of 18–24 weeks of gestation: a prospective cohort study

**DOI:** 10.1186/s12884-016-1021-x

**Published:** 2016-08-17

**Authors:** Kim M. J. Verdurmen, Carlijn Lempersz, Rik Vullings, Christian Schroer, Tammo Delhaas, Judith O. E. H. van Laar, S. Guid Oei

**Affiliations:** 1 Department of Obstetrics and Gynaecology, Máxima Medical Centre, P.O. box 7777, 5500 MB Veldhoven, The Netherlands; 2 Faculty of Electrical Engineering, Eindhoven University of Technology, P.O. box 513, 5612 AZ Eindhoven, The Netherlands; 3 Department of Paediatrics, Máxima Medical Centre, P.O. box 7777, 5500 MB Veldhoven, The Netherlands; 4 Department of Biomedical Engineering, CARIM School for Cardiovascular Diseases, Maastricht University, P.O. Box 616, 6200 MD Maastricht, The Netherlands

**Keywords:** Congenital heart disease, Fetal electrocardiography, Fetal electrocardiogram parameters

## Abstract

**Background:**

The fetal anomaly ultrasound only detects 65 to 81 % of the patients with congenital heart disease, making it the most common structural fetal anomaly of which a significant part is missed during prenatal life. Therefore, we need a reliable non-invasive diagnostic method which improves the predictive value for congenital heart diseases early in pregnancy. Fetal electrocardiography could be this desired diagnostic method. There are multiple technical challenges to overcome in the conduction of the fetal electrocardiogram. In addition, interpretation is difficult due to the organisation of the fetal circulation in utero. We want to establish the normal ranges and values of the fetal electrocardiogram parameters in healthy fetuses of 18 to 24 weeks of gestation.

**Methods/Design:**

Women with an uneventful singleton pregnancy between 18 and 24 weeks of gestation are asked to participate in this prospective cohort study. A certified and experienced sonographist performs the fetal anomaly scan. Subsequently, a fetal electrocardiogram recording is performed using dedicated signal processing methods. Measurements are performed at two institutes. We will include 300 participants to determine the normal values and 95 % confidence intervals of the fetal electrocardiogram parameters in a healthy fetus. We will evaluate the fetal heart rate, segment intervals, normalised amplitude and the fetal heart axis. Three months postpartum, we will evaluate if a newborn is healthy through a questionnaire.

**Discussion:**

Fetal electrocardiography could be a promising tool in the screening program for congenital heart diseases. The electrocardiogram is a depiction of the intimate relationship between the cardiac nerve conduction pathways and the structural morphology of the fetal heart, and therefore particularly suitable for the detection of secondary effects due to a congenital heart disease (hypotrophy, hypertrophy and conduction interruption).

## Background

During pregnancy, the condition of the fetus is assessed with different techniques. One of these techniques is ultrasound examination. Between week 18 and 22 of gestation the fetal anomaly ultrasound is performed. During this examination, the fetus is screened for all kind of possible congenital anomalies, including congenital heart disease (CHD). CHD is defined as a “gross structural abnormality of the heart or intra-thoracic large vessels, (possibly) with functional significance” [1]. CHD is the most common severe congenital anomaly worldwide [2], the incidence is estimated at 6–12 per 1000 live births [3–5]. CHD is six times more common than chromosomal anomalies and four times more common than neural tube defects [4, 6].

The fetal anomaly ultrasound, including planes of the ventricular outflow tracts and the three-vessel view, only detects 65 to 81 % of the patients with CHD [6–9]. That makes CHD the most common structural fetal abnormality of which a significant part is missed during prenatal life. Therefore, we need a reliable non-invasive diagnostic method which improves the predictive value for the diagnosis CHD. This diagnostic technique should be able to diagnose CHD early in pregnancy for multiple reasons. First, we get the opportunity to identify associated extracardiac and chromosomal anomalies that affect fetal and postnatal prognosis. Second, parents get the chance to opt for termination of pregnancy in case of a severe CHD. Third, one can develop an adequate treatment plan including intra-uterine therapy, timing, mode and location of delivery and planning of immediate treatment after birth. In ductus- and foramen ovale dependent CHDs, it is demonstrated that prenatal diagnosis increases the survival rates and decreases long term morbidity [10–13].

The currently used two-dimensional ultrasonography provides multiple anatomic planes, relying on the sonographists mental reconstruction of these planes to define the fetal cardiac anatomy. The antenatal diagnostic value is therefore to a great extent depending on the experience of the performer. As stated by Gardiner; “you only see what you look for and identify what you already know” [5]. Three- and four-dimensional ultrasonography gives a more fluid and representative image of the fetal cardiac structures, and therefore aids in this mental reconstruction [14]. Spatio-temporal image correlation (STIC) is a new modality using automated volume acquisition of the fetal heart with one sweep of the probe, further facilitating the examination of the fetal heart. Disadvantages of these ultrasound modalities are that they are extremely expensive and only applicable in centres with experienced personnel.

The non-invasive fetal electrocardiogram (ECG) could be a valuable tool for the detection of CHD early in pregnancy. In 1906, Cremer and colleagues were the first to describe fetal ECG extraction through the maternal abdomen [15] and 80 years later, Pardi and colleagues were the first to write a review considering fetal ECG and, amongst others, CHD [16]. Compared to other techniques for fetal monitoring, the development of the fetal ECG lagged behind. This is mainly because there are multiple technical challenges to overcome. First, at a gestational age of 20 weeks, the fetal heart is about 1/10th of the size of an adult heart. Due to the low voltage of the fetal ECG (1/50th  of the maternal ECG), there is a low signal-to-noise ratio. In addition, identifying the fetal signals is challenging due to masking by the maternal ECG and high background noises caused by the maternal electromyogram. The amniotic fluid and maternal tissues that surround the fetus enlarge the distance to the electrodes, and cause a non-homogenous tissue conduction that interferes with signal quality. In addition, the vernix caseosa causes electrical isolation and further diminishes the signal amplitude [17]. This is the main cause of the poor signal-to-noise ratio from 30 to 34 weeks of gestation [18, 19]. Second, the fetal ECG has a complex three-dimensional shape, alternating with changes in fetal presentation. Following fetal movements, the electrical signal from each electrode changes frequently. Another challenging factor is the speed of the fetal heart rate, which is two to three times faster compared to the adult heart rate [20].

Besides the technical difficulties encountered when conducting a fetal ECG, it is also challenging to interpret the fetal ECG. In contrast with postnatal life, the systemic circulation in the fetus is fed from both the left and right ventricle in parallel, with equal intraventricular pressures [21]. The outflow in the right ventricle is slightly larger compared to the outflow in the left ventricle, and increases during gestation; 53 % vs 47 % at 20 weeks of gestation, 57 % vs 43 % at 30 weeks of gestation and 60 % vs 40 % at 38 weeks of gestation [21]. In utero, the O_2_-rich blood flows from the umbilical vein to the right atrium. There, the formamen ovale propels a major part of the O_2_-rich blood to the left side of the heart and into the systemic circulation, bypassing the fetal lungs. In addition, in the second trimester the ductus arteriosus propels 40 % of the combined cardiac output. Because of these major differences between the systemic circulation in utero and postpartum, it is difficult to predict what a normal fetal ECG looks like. Furthermore, due to this organisation of the fetal circulation in utero, in case of a CHD one side of the heart can compensate for an abnormality on the other site, and fetuses affected by a CHD do not always show signs of cardiac failure.

However, before we are able to detect CHD with the fetal ECG, we need to establish the normal range and values of amplitudes and segment intervals of the fetal ECG in a healthy fetus.

## Methods/Design

### Aim

The aim of this study is to establish the normal ranges and values (mean with 95 % confidence intervals) of fetal ECG parameters in a healthy fetus of 18 to 24 weeks of gestation.

### Study design

We will perform a prospective cohort study. The study protocol is approved by the medical ethical committee of the Máxima Medical Centre, Veldhoven, the Netherlands (NL48535.015.14).

### Setting

Measurements are performed at the Máxima Medical Centre, Veldhoven, the Netherlands and “Diagnostiek voor U” (DvU), Eindhoven, the Netherlands. The Máxima Medical Centre is a tertiary care teaching hospital for obstetrics. DvU is a diagnostic centre which, amongst others, performs blood tests and ultrasounds. Measurements are performed directly before or after the sonographist performed the fetal anomaly scan. The fetal anomaly ultrasound is performed by a certified and experienced sonographist.

### Participants

Patients with an uneventful pregnancy, carrying a singleton fetus with a gestational age between 18 and 24 weeks, are included in the study after written informed consent. At the Máxima Medical Centre, this will be patients who visit the outpatient clinic for an appointment. At DvU, this will be patients who visit the centre for their fetal anomaly ultrasound. These patients are generally seen by a midwife or by a doctor at the Máxima Medical Centre for their obstetrical care. Pregnant women must be aged older than 18 years. If any of the fetuses turn out to have a form of CHD, they are excluded from the cohort. Other exclusion criteria are multiple pregnancies, insufficient understanding of the Dutch language, and any known fetal congenital anomalies.

Three months postpartum we will evaluate if the newborn is healthy, which is defined as absence of CHD, through a questionnaire. If the neonate turns out to have a CHD, which was missed at the time of the structural anomaly ultrasound, we will exclude the patient from the cohort.

### Procedures

**Fig. 1:**
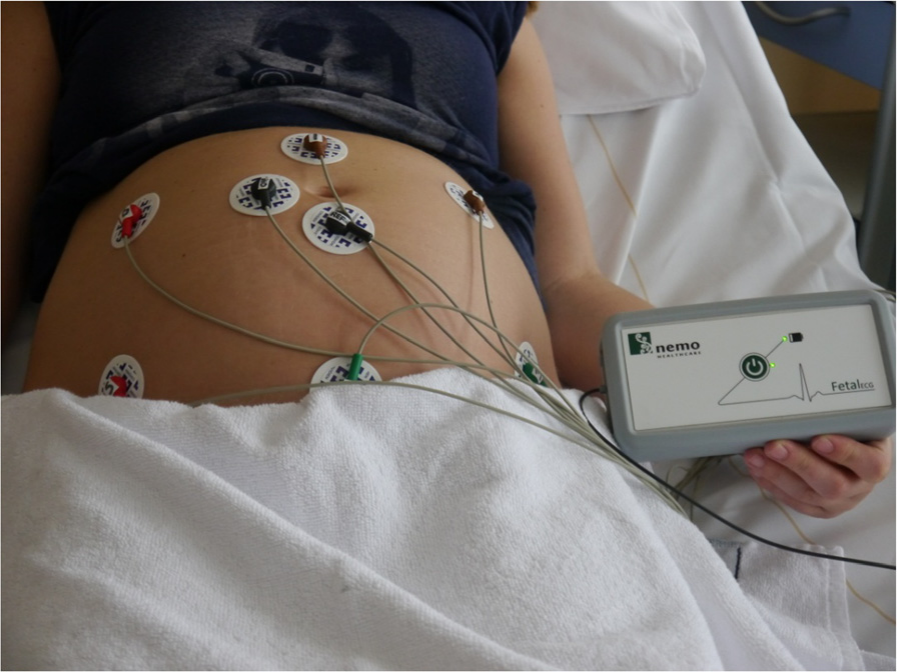
Configuration of the electrodes on the maternal abdomen. The fetal ECG is recorded with eight electrodes on the maternal abdomen, placed in a fixed configuration. A ground and reference electrode are placed near the belly. The electrodes are connected to our fetal ECG system, which is connected to a computer. This system records six channels of fetal ECG data

**Fig. 2:**
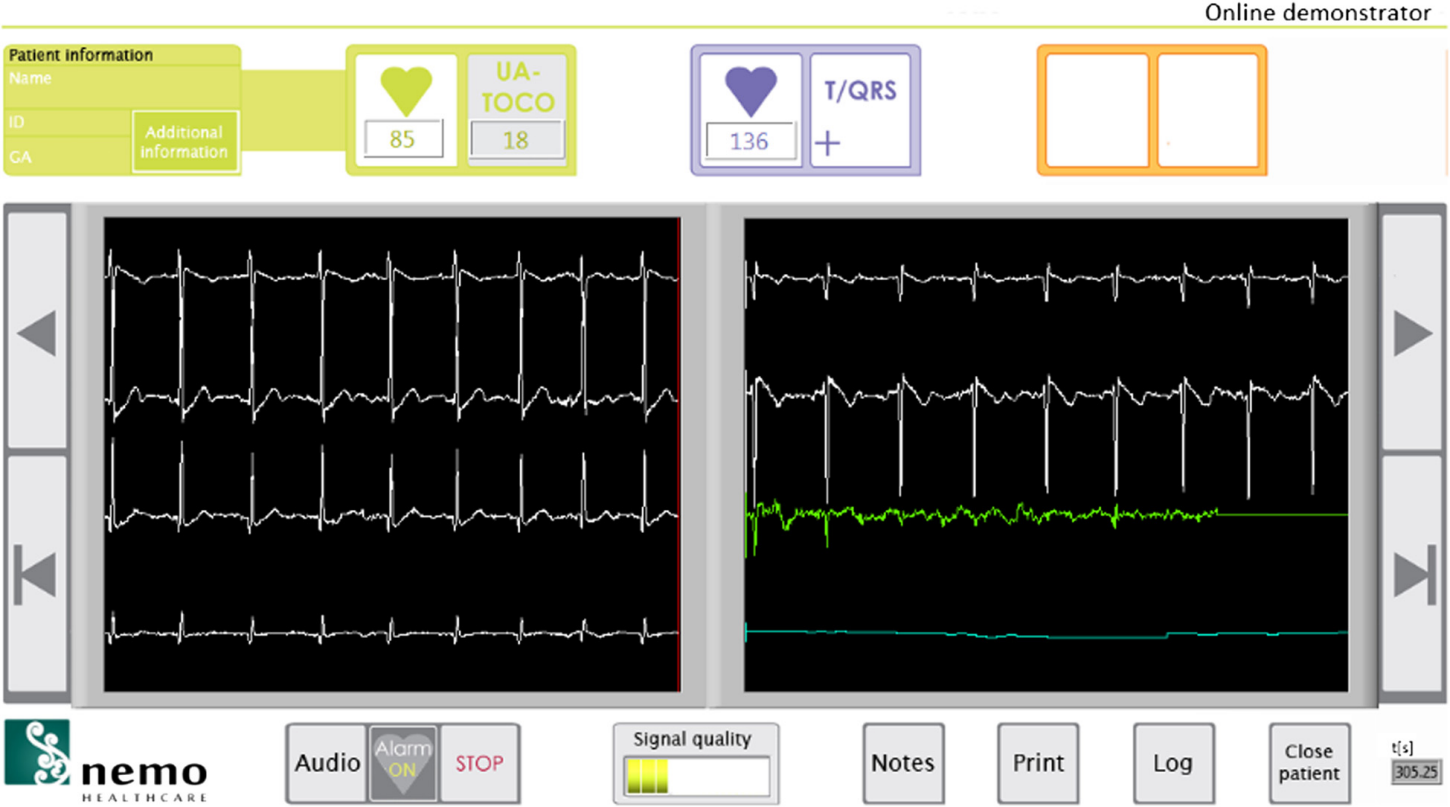
Real-time bedside monitoring system. Good signal quality can be verified via the real-time bedside monitoring system. Below the *green heart* in the *top panel* on the left, the maternal heart rate is depicted. Right next to this, the uterine activity is shown. Below the *blue heart* on the right side of the *top panel*, the fetal heart rate is depicted. The *white lines* represent the output from the six abdominal electrodes, while the *green line* is a computation of the fetal signal, after subtraction of the maternal signal. In the *lower panel* in the middle, an estimate of the signal quality is shown. The user interface can be switched to a different screen in which the cardiotocogram is depicted

The fetal ECG is a non-invasive, transabdominal research method. The pregnant women is lying down in a semi-recumbent position to prevent aortocaval compression. The fetal ECG is conducted with eight electrodes on the maternal abdomen, placed in a fixed configuration (Fig. 1; consent for publication is obtained). Before applying the electrodes on the abdomen, the skin is cleaned and prepared by scrubbing the skin areas with abrasive paper to optimise the skin-electrode impedance. The impedance is measured after the skin is prepared and before the fetal ECG recording is started. A ground and reference electrode are placed near the belly. The six recording electrodes give bipolar signals that, among others signals, contain the fetal ECG. The placement of the electrodes is chosen in order to assess the fetal heart with as much accuracy as possible. With the fetus able to move freely in the uterus, at least some of the six electrodes will be close to the fetal heart and thus will give a usable bipolar signal. We will record the fetal ECG for 30 min. During this recording, the fetal position is determined four times by ultrasound assessment. Good signal quality is verified via the real-time bedside monitoring system (Fig. 2).

The fetal ECG signals are digitised and stored by a prototype fetal ECG system (NEMO Healthcare BV, the Netherlands). This prototype system comprises of a 6-channel amplifier that is dedicated for electrophysiological recordings during pregnancy. After digitization, the acquired signals are processed by PC-based dedicated signal processing techniques as previously described by Vullings et al. [22, 23] to suppress interferences such as maternal ECG, powerline, and electromyographic signals from within the maternal body, and retrieve the fetal ECG. Following, we can calculate the fetal ECG for each of the six electrodes. However, before we can compare ECG values between patients we need to normalise the ECG for different orientations of the fetus within the uterus. A specific electrode would record a different ECG waveform for a fetus in cephalic position versus for a fetus in breech position, also yielding differences in some of the ECG parameters mentioned below.

To normalise for fetal orientation, we calculate the vectorcardiogram (VCG) of the fetus [24]. This VCG entails a 3-dimensional representation of the fetal electrical cardiac activity. As described by Frank et al. [25], in adult electrocardiography the VCG can be used to calculate standardised ECG leads such as Einthoven 1–3, aVF, aVL, and aVR. By mathematically rotating the fetal VCG prior to calculating the ECG, we can create standardised fetal ECG leads. The amount of rotation required is determined based on a simultaneously performed ultrasound assessment of the fetal presentation. Via these mathematical rotations, we are also capable of detecting and correcting for fetal movements in between the ultrasound assessments, as described previously by Vullings et al. [26]. The four ultrasound assessments during the measurements are used to correct for cumulative errors in this correction method and to determine the initial orientation of the fetus. To enhance the signal quality of the measurements, the fetal ECG is filtered further (amongst others by averaging of the ECG waveforms). The detection of segments and intervals is performed semi-automatically. The detection of fetal ECG complexes is computerised, while marking of the fetal ECG intervals (P top, QRS complex, T top) is performed manually by two independent researchers following a protocol that is verified by an experienced paediatric cardiologist. We will calculate the inter-observer variability between the two researchers.

Normal heart rhythm is assumed to show variations in heartbeat intervals smaller than 20 % between consecutive beats. In case these variations are larger, this is assumed to be the result of either fetal arrhythmia or erroneous detection of the heartbeat interval, e.g. because of poor signal quality. Assessment of erroneous detection is based on energy of the ECG signal and correlations between consecutive ECG waveforms. The ECG is a quasi-periodic signal, meaning that consecutive ECG waveforms have a similar appearance and similar amplitude/energy. In case of poor ECG signal quality, the energy of the ECG signals is expected to differ from the energy during good quality recordings. Present artefacts or noise cause the energy of the ECG to increase beyond physiologically plausible ranges. Likewise, correlations between consecutive ECG waveforms are reduced in the presence of poor signal quality.

It has to be noted here, that fetal arrhythmia can also cause poor correlation between ECG waveforms. Some arrhythmias are hence expected to be incorrectly classified as poor signal quality. This misclassification affects the detection of fetal arrhythmia, but will not have any impact on other study parameters as these are determined only during normal rhythm and good signal quality. The recording must contain a minimum of 200 ECG complexes that were assessed to have good signal quality and that were corrected for fetal movement [27].

### Study parameters

Multiple outcome values are evaluated:

- Fetal heart rate; mean, standard deviation, 95 % confidence intervals and heart rate arrhythmia
- Segment intervals (PQ, QRS, ST etc.); mean, standard deviation and 95 % confidence intervals
- Normalised amplitudes (P, QRS, T); mean, standard deviation and 95 % confidence intervals
- Fetal electrical heart axis
- % of total patients in which the recording contains the required amount of data to perform the analysis

Heart rate arrhythmia is defined based on heuristic rules that dictate that during normal rhythm subsequent heartbeat intervals cannot differ more than 20 %. Any rhythm not complying with this rule, and assessed to not be caused by erroneous detection of heartbeats, e.g. as a result of poor signal quality, is labelled as a fetal arrhythmia.

### Sample size

There are previously published studies (see Discussion for more details) that describe fetal ECG parameters. However, these studies use different methods for obtaining the fetal signal and do not correct for the fetal position in the uterus. Therefore they are not able to calculate the fetal electrical heart axis. Moreover, all studies describe another parameter of the fetal ECG. Statistical experts calculated that we need a study population of 200 pregnant patients in order to determine normal values and 95 % confidence intervals of a healthy fetus [22]. Anticipating on loss to follow-up and insufficient data quality, we will include 300 patients in the initial cohort.

### Statistical analysis

The collected data is analysed through SPSS. With the collected data, we perform several analyses. We calculate the normal values and ranges of the fetal heart rate, segment intervals (PQ, QRS, ST etc.), normalised amplitudes (P, QRS and T) and the fetal heart axis. Initially, we will calculate the values and ranges for all included patients as one group (18 to 24 weeks of gestational age). Thereafter, we will perform a subanalysis for every group per week of gestational age.

## Discussion

Previous studies have been published regarding the normal values and ranges of the fetal ECG. In their review, Pardi et al. summarized the normal evolution of the cardiac cycle during gestation [16]. From the 17th week of gestation up to term, the duration of the P-wave increases progressively. This reflects the anatomical development of the atria during pregnancy. Similar, the duration of the QRS-complex increases progressively, parallel with the weight gain of the fetal heart and in particular with the gain in ventricular mass. In fetal life, the intraventricular conduction is delayed compared to adult values, most likely due to anatomical differences of the ventricular conduction tissue. There is a slight increase in PR-interval during pregnancy, indicating development of the atrioventricular conduction tissue.

Recently, longitudinal studies followed pregnancies from 14 to 41 weeks of pregnancy and performed fetal ECG measurements during different stages of gestation [18, 23, 24]. In the 1960’s, Larks and colleagues described the orientation of the electrical fetal heart axis [25, 26, 28, 29]. All mentioned studies performed fetal ECG recordings with different conduction and analysis techniques. The amount of electrodes on the maternal abdomen varied from three to sixteen. Average fetal ECG complexes were generated from segments of 60 s up to 2.5 min. Analyses were performed manually or by computerized signal processing programs. These studies did not take the fetal position in the uterus into account.

In a group around 20 weeks of gestation, the following mean values were found by Chia and Taylor respectively: P wave length 43.9 ms, PR interval 102.1/91.7 ms, QRS duration 47.2/40.7 ms, QT interval 224.0/242.3 ms and T wave duration 123.8 ms [23, 24]. Larks found a normal range of the fetal heart axis between +100 and +160°, with a mean value of +134° in term fetuses during labour [29]. Due to the lack of correcting for the fetal position in utero, fetuses in breech position showed a negative electrical heart axis (−180 to 0°) [28]. In fact, due to the lack of correcting for fetal position, also findings for fetuses in vertex position were unreliable. In their analysis, Larks implicitly assumed that every fetus was facing the frontal plane. In cases where this assumption was incorrect, the measured heart axis must have been incorrect as a consequence. For example, a fetus with an electrical heart axis at +135° will indeed be measured as +135° when facing the frontal plane. When this same fetus, still in vertex position, rotates to face the sagittal plane, the measured heart axis will be +90°. When opposing the frontal plane, the measured heart axis will be +45°.

Up to our knowledge our study is the first to calculate the fetal electrical heart axis, taken the fetal position into account. A reliable calculation of the electrical heart axis is important in interpreting the fetal ECG. In addition, changes in the orientation of the fetal electrical heart axis might be able to aid in the diagnosis of congenital heart disease in the future.

The fetal ECG can be used from early gestation, it is non-invasive, easy to apply and safe to use [18]. One of the big advantages of the fetal ECG is that it potentially is a non-expensive diagnostic test in the long term. In addition, it creates the opportunity to perform measurements anywhere in the world and transmit the raw ECG data to be evaluated elsewhere. The equipment is smaller in comparison to ultrasound machines. Moreover, the fetal ECG is evaluated by semi-computerized algorithms, taking away some of the performer-dependent variability in diagnostic value. The fetal ECG system takes minimum training to be applied.

The fetal ECG could be a promising clinical tool in the screening program for CHD. It is a depiction of the intimate relationship between the cardiac nerve conduction pathways and the structural morphology of the fetal heart [8, 30]. The fetal ECG is likely to be particularly suitable for the detection of secondary effects due to a CHD; hypotrophy, hypertrophy and conduction interruption.

## Abbreviations

CHD, congential heart disease; ECG, electrocardiograhy/electrocardiogram; STIC, spatio-temporal image correlation
